# E-cigarettes: online survey of UK smoking cessation practitioners

**DOI:** 10.1186/1617-9625-12-13

**Published:** 2014-08-21

**Authors:** Rosemary Hiscock, Maciej Lukasz Goniewicz, Andy McEwen, Susan Murray, Deborah Arnott, Martin Dockrell, Linda Bauld

**Affiliations:** 1 Department for Health, University of Bath, Bath, UK; 2 UK Centre for Tobacco and Alcohol Studies, Nottingham, UK; 3 Department of Health Behavior, Roswell Park Cancer Institute, Buffalo, USA; 4 National Centre for Smoking Cessation and Training (NCSCT), London, UK; 5 Cancer Research UK Health Behaviour Research Centre, University College London, London, UK; 6 School of Applied Social Science, University of Stirling, Stirling, UK; 7 Action on Smoking and Health, London, UK; 8 Public Health England, London, UK; 9 Institute for Social Marketing, University of Stirling, Stirling, UK

**Keywords:** E-cigarettes, NHS stop smoking services, Practitioners

## Abstract

**Background:**

Use of e-cigarettes (inhalable vapour producing battery powered devices that aim to simulate tobacco cigarettes), is rising in a number of countries, but as yet none of these products are regulated as medicinal devices or available as smoking cessation treatments. Smokers seeking support from health professionals to stop smoking are interested in e-cigarettes and may be buying them to aid a quit attempt. Determining what smokers are asking, and what health professionals think about these products may have implications for smoking treatment services in a number of countries.

**Methods:**

Stop smoking service advisors, managers and commissioners in the United Kingdom were asked to take part in two surveys on e-cigarettes. Data was analysed from 587 practitioners who completed a survey in 2011 and 705 practitioners who completed a repeat survey in 2013. Responses to multiple choice questions and free text comments were analysed.

**Results:**

Responding practitioners reported that interest in, and use of, e-cigarettes is growing among adults seeking help to stop smoking in the UK. In 2013 91% of respondents reported that interest in e-cigarettes had grown in the past year and whilst in 2011, 2% of respondents reported a ‘quarter to a half’ of their clients saying that they were regularly using e-cigarettes, by 2013 this had increased to 23.5% (p < .001). Responding practitioners’ views towards e-cigarettes became more positive between the first and second surveys (15% strongly agreed/agreed in 2011 that ‘e-cigarettes are a good thing’ rising to 26% in 2013). However, they continued to have concerns about the products. In particular, analysis of free text responses suggested practitioners were unsure about safety or efficacy for smoking cessation, and were worried that smokers may become dependent on the products. Practitioners were also aware of the potential of e-cigarettes to undermine smokers’ willingness to use evidence-based methods to stop, and to challenge policies aiming to denormalise tobacco smoking.

**Conclusions:**

Health professionals are asking for reliable and accurate information on e-cigarettes to convey to smokers who want to quit. Randomized controlled trials and ongoing surveillance of e-cigarette use and its consequences for smoking cessation rates and smoking treatment services are required.

## Introduction

E-cigarettes are battery powered devices that aim to simulate tobacco cigarettes by heating nicotine and other chemicals into an inhalable vapour. Use of e-cigarettes has grown rapidly in recent years although their availability differs between countries. Estimates from the International Tobacco Control Survey conducted in US, Canada, Australia and UK, suggest that 2.9% of current and ex-smokers in 2010 were users of e-cigarettes, 7.6% had tried e-cigarettes and 46.6% were aware of them [1]. In the UK, it is estimated that there were 600,000 current users in 2012 and that use more than doubled from 2.7% of the population in 2010 to 6.7% in 2012 [2]. Current estimates suggest that in 2014 there are well in excess of two million users [3]. In line with this increase in use, a growing body of research on these products exists, but some types of evidence on their safety and efficacy for smoking cessation remain limited [4-6].

E-cigarettes usually provide nicotine and potentially act as a behavioural replacement for smoking [7,8]. Although they usually deliver less nicotine than cigarettes, they have been found to alleviate craving and cigarette withdrawal symptoms [9-11]. Several surveys report that e-cigarette users consider the product a satisfactory replacement for cigarettes and an effective stop-smoking treatment [12-15]. It is therefore not surprising that health professionals who support smokers to stop are being asked to provide information on e-cigarettes [16]. However, no country has yet licensed e-cigarettes as medicines and they are not available anywhere on prescription [17-19].

In the UK, the Medicines Healthcare Regulatory Agency (MHRA) announced in June 2013 that e-cigarettes manufacturers would be required to seek a medicinal license if their products contained nicotine and were intended to act as a cessation aid from 2016 [20]. However, this decision was to be linked to European legislation (the EU Tobacco Products Directive) and this legislation now looks likely to permit many e-cigarettes to continue to be sold as consumer products in Europe although manufacturers will need to provide better quality information on safety and quality [21].

In the UK, a small proportion of smokers are willing to access the UK national Stop Smoking Service (SSS) for help with their quit attempt (around 5% of those making a quit attempt with pharmacological or behavioural support) but this still amounts to over 700,000 clients each year [22,23]. Other surveys have looked at how SSS are responding to the increased use of e-cigarettes and have found that the use of e-cigarettes by clients of SSS is common, but that there is little or no systematic recording of their use [16].

SSS clients and staff are an important group to study for a number of reasons. First a growing number of countries around the world are offering smoking cessation services [24] and clients and staff in some of these other countries may have similar questions about e-cigarettes. Such staff are credible sources of information for smokers and are trained to accurately describe stop smoking medications to clients [25], thus any concerns they have over answering clients’ queries about a new product should be addressed. Secondly smokers attending the UK services have a greater chance of quitting than smokers attempting to stop by using medication or willpower alone [26], and the reported experience of smokers already willing to use effective cessation aids regarding e-cigarettes may be particularly valuable. Finally, the views of service staff on e-cigarettes can be useful for policy makers in considering how these products may impact upon the services and medication currently provided by SSS and whether e-cigarettes may or may not have a place in tobacco harm reduction, as staff could be tasked with implementing new policies on e-cigarettes in the future.

This study’s objective was to explore the extent to which smokers seeking help to stop smoking with the SSS were asking about e-cigarettes, the reported usage of e-cigarettes by clients, the concerns clients presented about e-cigarettes and what the views of practitioners were regarding these products. Within the study there is a primary source of information – practitioners’ opinions of e-cigarettes, and also a secondary source of information – practitioners’ reports of their clients’ experience of e-cigarettes.

Two cross-sectional surveys were conducted in 2011 and 2013 with smoking cessation practitioners working in a SSS. This was a period when national and European policy on e-cigarettes was gradually developing and so an important interval within which to track any changes over time. The surveys were designed to assess the following:

•whether queries about and use of e-cigarettes changed between the two surveys,

•what types of queries were being received in 2011 and 2013,

•the extent to which practitioners perceived e-cigarettes as a positive or negative development and whether this changed over time,

•needs for more research and guidance and the assessment of current guidance.

## Methods

### Setting and procedures

In 2011 the authors developed a short questionnaire on e-cigarettes for SSS practitioners, following a discussion on the topic at a national conference. The questionnaire was piloted with two practitioners and then developed into an online survey using Bristol Online Surveys [27]. In June 2011 a link to this survey was sent by the National Centre for Smoking Cessation and Training (NCSCT), the main body for cessation training in England, to all SSS practitioners registered with them. These practitioners were staff engaged in supporting smokers to stop or managing staff who supported smokers to stop (generally employed by specialist stop smoking services, GP practices and pharmacies). Responses were received between June and August 2011, with one reminder issued in July. In June 2013, the survey was reviewed and slightly revised by the study team with a view to assessing any changes in opinions amongst practitioners in the intervening period. A link to this second survey was sent to practitioners on the same list and responses were received in July 2013, with no reminders required as the volume of responses received was large. The surveys took less than five minutes to complete. As it was voluntary, reviewed current service provision and the contact list was held by a national training body, NHS ethics approval was not required. It was possible for practitioners to complete both surveys.

### Measures

Four topics were covered by the two surveys: first, the extent of clients’ queries about e-cigarettes and practitioners’ estimates of prevalence of use among their clients (ever use and regular use) (see Table 1 for response options); secondly the types of queries that practitioners received from clients. Respondents were asked what types of questions they had received about e-cigarettes and were provided with a list of twelve potential client queries: where to get them, how much they cost, whether they were legal, whether SSS provide them and why SSS don’t provide them, whether they contained harmful additives, how safe they are for users or others around them, whether they were effective for stopping smoking or cutting down or avoiding smoking, how they function and any other problems with the product. In 2011 respondents were able to choose one of these queries and in 2013 respondents could choose as many queries as they liked. Thirdly the practitioners’ own views on e-cigarettes were elicited through asking, using a five point Likert scale (see Table 1 for response options), to indicate the extent they agreed that “E-cigarettes are a good thing”. In addition respondents were able to leave free-text comments on e-cigarettes or their use by clients which provided a more nuanced response. Fourthly practitioners’ needs for further guidance on e-cigarettes and their assessment of current available guidance in the UK on e-cigarettes were assessed. In the 2011 survey practitioners were asked if they wanted more information and guidance on e-cigarettes using a Likert scale (see Table 2 for response options). In 2013 practitioners were asked whether they knew about the recent MHRA guidance that had been published just before the second survey and whether they thought it was useful using a variety of response scales (see Table 2 for response options). These questions were derived specifically for this study.

**Table 1:** Queries about, use of and opinions on e-cigarettes among clients as reported by SSS practitioners

	**2011 survey**		**2013 survey**		
	**N**	***%***	**N**	***%***	***p***
**Clients asking about e-cigs** **compared to one year ago**					*P < .001*
More clients asking	338	*64.3*	607	*90.7*	
Same proportion	129	*24.5*	48	*7.2*	
Fewer clients asking	59	*11.2*	14	*2.1*	
Total	526	*100.0*	669	*100.0*	
**% clients ever used e-cigs**					*P < .001*
None	50	*9.9*	13	*2.0*	
Less than a quarter	404	*79.8*	283	*43.2*	
Quarter to a half	38	*7.5*	262	*40.0*	
Half to three quarters	6	*1.2*	80	*12.2*	
More than three quarters	8	*1.6*	17	*2.6*	
Total	506	*100.0*	655	*100.0*	
**% clients regularly using e-cigs**					*P < .001*
None	90	*18.6*	25	*3.8*	
Less than a quarter	382	*79.1*	445	*67.8*	
Quarter to a half	11	*2.3*	154	*23.5*	
Half to three quarters	0	*0.0*	23	*3.5*	
More than three quarters	0	*0.0*	9	*1.4*	
Total^1^	483	*100.0*	656	*100.0*	
**Clients queries about e-cigs**		*P < .001*		*P < .001*	
Where to get them?	249	*49.2*	227	*6.1*	
Do SSS provide them?	4	*0.8*	569	*15.4*	
Why don’t SSS provide?	36	*7.1*	434	*11.8*	
How much do they cost?	3	*0.6*	188	*5.1*	
Are they legal?	0	*0.0*	138	*3.7*	
Contain harmful additives?	63	*12.5*	356	*9.6*	
Safe for users?	67	*13.2*	436	*11.8*	
Safe for OTHERS around?	2	*0.4*	122	*3.3*	
Effective for stopping?	68	*13.4*	514	*13.9*	
Effective for cutting down?	2	*0.4*	341	*9.2*	
Problems with products?	1	*0.2*	100	*2.7*	
How they work?	11	*2.2*	267	*7.2*	
Total queries^1^	506	*100.0*	3692	*100.0*	
**‘E-cigs are a good thing’**					*P < .001*
strongly agree	27	*4.6*	47	*6.7*	
agree	60	*10.2*	135	*19.2*	
unsure	282	*48.0*	293	*41.6*	
disagree	123	*21.0*	94	*13.3*	
strongly disagree	95	*16.2*	106	*15.0*	
Total	587	*100.0*	675	*100.0*	

^1^Totals for clients’ queries analysis refer to number of queries.

**Table 2:** Summary of comments from SSS practitioners in 2011 (n = 174 comments) and 2013 (n = 263 comments)

	***% 2011 survey comments***	***% 2013 survey comments***	***Change***	
*Positive comments*	*38*	*40*	*+2*	
Popular	8	10	*+*2	“it is very popular. It’s no good ignoring it…it’s here to stay”
Help with quitting	9	12	*+*3	“the majority found them successful in helping them to quit”
Reduce harm	9	13	*+*3	“A good, harmless e.cig has to be better than smoking”
Very like cigs- cf NRT	12	6	−6	“All of my clients found e cigarettes a lot better than using inhalators. They felt it was more like smoking”
*Negative comments*	*49*	*69*	*+20*	
Do not help quitting	20	12	−8	“Anything that still looks and feels like a cigarette is not adequately breaking of the old habits”
Operational issues	10	6	−4	“Several clients have relapsed when they ran out of refills for device or could not wait for it to charge”
Safety (inc dependence)	11	24	*+*13	“like giving Heroin addicts Methadone” “they seem to be as addictive (if not more) than cigarettes”
Undermines other tobacco control measures	9	27	*+*19	Prevention: “Children and young people see the device as fun”
				Marketing bans: “they are put in packs like cigarettes and allowed to place advertisements”
				Pharmacotherapy: “A client..quit for 6 weeks using the inhalator …He purchased an e-cig… and found that he could get the “hit” that cigarettes used to give him. This lead to increased strong cravings and he eventually returned to smoking. If he had continued with the slow, gentle levels of nicotine from the inhalator I feel he would have been more likely to stay quit”
				SSS: “They are reducing the numbers… accessing the evidence based smoking cessation services”
				Smokefree: “I feel it undermines the denormalisation of smoking and confuses the SF policies I write”
*Research/guidance needs*	*51*	*55*	*+4*	
Need more info/guidelines	28	24	−5	“My organisation refuses to rule or take a stance on them so advisors are left in a limbo”
Companies involved	21	14	−7	“I feel young people will become addicted to nicotine by using them and big tobacco is quids in!!”
Probs with current guidelines	1	9	*+*9	“How can the MHRA state that no e-cigs tested by them would be licensed yet make no move to restrict their sale for 3 years?”
Integration with SSS	1	8	*+*7	Pro: “I feel that any ‘safer’ alternative to smoking should be available to clients through NHS services”
				Anti:
				“We are a Stop Smoking Service, and we have to remember that is the aim, to help people stop smoking”
				“The ability to deliver the harm reduction [NICE] guidance on tight tobacco control budgets is… a concern”

Note that comments could include positive, negative and research/guidance needs statements so percentages do not sum to 100.

### Analysis

Stata [28] was used to explore prevalence of use and queries and attitudes towards e-cigarettes. Chi square tests were used to compare differences between the 2011 and 2013 surveys. Responses to questions about information and guidance were dichotomised into ‘yes’ or ‘no’, ‘true’ or ‘false’ or ‘strongly agree’ and ‘agree’ compared with ‘neutral’, ‘disagree’ and ‘strongly disagree’. Percentages for the dichotomies were calculated and tabulated.

For the analysis of query type, counts refer to the number of queries rather than the number of respondents: in 2011 each respondent could choose one of 12 query types and 506 respondents indicated a query type so there were 506 queries. In 2013 respondents could choose as many of the 12 query types as they liked. A total of 3692 queries were indicated (on average 5 of the 12 query types were indicated by each practitioner). We could not directly compare between years because in 2011 respondents only indicated one query type whereas in 2013 respondents could indicate as many as required.

Comments were transposed verbatim and analysed separately. A coding frame was devised for the 2011 survey. As this is a new area codes were generated from the data. Initially each comment was categorised as belonging to one or more of three macro codes ‘positive stance on e-cigarettes’, ‘negative stance on e-cigarettes’ and ‘information/guidance requirements’. The data in each of these macro codes were then divided into topics and, when required, subtopics. When the 2013 verbatim data became available comments were coded into the existing codes when possible and new codes and subcodes were added where necessary. The coding frame was initially developed by one author and then revised and developed by another author with input from a third author. The number of comments in each code were summed and converted to a proportion of the total comments from each survey (see Additional file 1).

## Results

In 2011, 587 out of 3075 practitioners registered with the NCSCT responded and in 2013 705 out of 20 024 registered practitioners responded. Note that the survey period was shorter in 2013 as the desired sample size was achieved more quickly. In 2011 85% were advisors, 8% were managers and 4% were commissioners and 4% were ‘other’ and in 2013 78% were advisors, 8% were managers and 2% were commissioners. In 2011, 174 practitioners entered comments and 263 did so in the 2013 survey.

### Prevalence of interest in and use of e-cigarettes among SSS clients

There was a significant increase in reported enquiries from clients. In 2011 about two thirds (64%) of respondents said more clients were asking about e-cigarettes, by 2013 this had risen to 91% (p < .001, Table 1). The proportion of clients reported by respondents as ever having used an e-cigarette was also higher in the 2013 survey (p < .001): in 2011 the majority (80%) said less than a quarter of their clients had reported ever using an e-cigarette, by 2013 this had dropped to 43% but the percentage reporting that a quarter to a half of clients had ever used e-cigarettes rose from 8% in 2011 to 40% in 2013. Reports of regular use of e-cigarettes also significantly increased: in 2011, 2% of respondents reported a ‘quarter to a half’ of their clients saying that they were regularly using e-cigarettes, by 2013 this had increased to 24% (p < .001).

### The nature of clients’ queries

In 2011 nearly half of queries mentioned concerned ‘where to get them’ (49%). The most common questions in 2013 (representing more than 10% of answers) were ‘do SSS provide them’ (15%), ‘why don’t SSS provide them’ (12%), ‘are they safe for users’ (12%) and ‘are they effective for stopping smoking’ (14%).

### Positive and negative views of e-cigarettes among practitioners

Practitioner opinion on whether e-cigarettes were ‘a good thing’ shifted significantly within the two years (p < .001). Despite around half still saying they were unsure about these products (48% in 2011 and 42% in 2013), there was more support for the view that e-cigarettes were a good thing (15% agreed (or strongly agreed) in 2011 rising to 26% in 2013, coupled with a 9% drop in those who disagreed (from 21% to 13%) between 2011 and 2013.

Many of the comments also addressed whether responding practitioners had a positive or negative opinion of e-cigarettes (Table 2). Analysis of positive comments (such as their popularity, success in helping with smoking cessation and harm reduction) indicated little change between 2011 and 2013. A 2011 comment stated “they are very good aid to quit smoking” and a 2013 comment stated “I think that e-cig are very good news”. However, a larger proportion of the comments in the later survey indicated that respondents had growing concerns about the safety of e-cigarettes, particularly about clients becoming dependent on the products (11% in 2011 compared with 24% in 2013). For example one of the seven 2011 comments mentioning dependence was:

“I recently saw a patient who had become addicted to the ‘e-cigarette’. He was using approximately 55 mg of Nicotine per day and was unable to leave the house without a supply… I sincerely hope that this is not the tip of the iceberg”.

In 2013 one of the 41 comments that mentioned dependence was:

“over the last few month[s] more clients are coming to the stop smoking clinics saying they have tried e-cigarettes but they still wanted to smoke as soon as they didn[‘] t use them”.

Thus dependence appeared, to respondents, to be an emerging issue with e-cigarettes.

Another concern was the possibility that e-cigarettes might undermine other tobacco control measures (9% in 2011 compared with 27% in 2013). For example, respondents expressed concern that fewer smokers were using stop smoking services or other evidence-based smoking treatment options in favour of using e-cigarettes. One practitioner suggested: “A lot of people are now avoiding accessing services as we do not supply e cigarettes.” Some were worried that e-cigarette advertising could contribute to ‘re-normalising’ smoking or undermine the enforcement of smokefree legislation for example one practitioner commented “[Local] schools… have had disruptions in exam and classroom enviro[n]ments as young people are.. showing tutors the packets where it states ‘safe to use’”. Guidelines are needed to enable the SSS to provide advice to such institutions used by young people.

### Respondents’ views of available guidance and information on e-cigarettes

Over 90% of respondents agreed that more research and information were needed in 2011 (Table 3). In 2013, nearly 90% had heard about the decision in the UK by the MHRA to pursue regulation of e-cigarettes as medicines from 2016 and 80% agreed (or strongly agreed) that this was a positive step. The majority were also aware of the limitations of the MHRA’s decision with less than a fifth believing that the decision enabled all e-cigarettes to be licensed and only 16% agreeing that new novel devices will be denied to smokers. There was also confusion about the implications of the MHRAs move for e-cigarette marketing with the respondents equally divided over whether or not marketing to children would be prohibited. Only 7% agreed with tobacco companies selling e-cigarettes implying that most responding practitioners would welcome further regulation in this direction.

**Table 3:** Practitioners opinions of available guidance on e-cigarettes

	**%**
**2011**	
Need more information (strongly agree/agree* vs neutral, disagree, strongly disagree)	90.2
Need more research (*strongly agree/agree* vs neutral, disagree, strongly disagree)	91.0
**2013**	
Do you know about the MHRA announcement about regulating e-cigarettes? (*yes* vs no)**	86.6
The MHRA decision to regulate e-cigarettes is a positive step for public health (*strongly agree/agree* vs neutral, disagree, strongly disagree)	81.3
The MHRA decision means that all e-cigarettes are now licensed by the MHRA (*true* vs false)**	18.7
The MHRA decision to regulate e-cigarettes will deny smokers access to new novel nicotine devices (*strongly agree/agree* vs neutral, disagree, strongly disagree)	16.3
MHRA regulation of e-cigarettes will prohibit marketing to children (*true* vs false)**	51.4
How do you feel about companies that are subsidiaries of tobacco companies bringing medications to market? (*strongly agree/agree* vs neutral, disagree, strongly disagree)	6.7

*Percentages of practitioners whose answer was in the italicised categories are tabulated.

**Only includes responses from English practitioners (n = 619) where MHRA guidance applies directly.

Despite the MHRA announcement and accompanying published research from a range of countries on e-cigarette products in 2013, there were a similar proportion of open-ended comments in both surveys requesting more research and clearer information for health professionals in terms of what they could say to their clients about these products (51% in 2011 and 55% in 2013) (Table 2). One advisor in 2013 voiced these concerns: “[we appear] impotent to the issue…: unable to recommend the products or not” In the 2013 survey there were also questions about how best to support smokers who were trying to stop while using e-cigarettes alongside licensed medicines such as Nicotine Replacement Therapy (NRT) and concerns about the delay in licensing any e-cigarettes (for example: “a safe licensed e-cigarette that stop smoking services can recommend/prescribe is definitely needed in the near future”) so that they could be prescribed to smokers trying to quit.

## Discussion

To our knowledge this is the first published paper to report on repeat surveys of smoking cessation practitioners and their clients’ reported use of, and concerns about, e-cigarettes. The findings highlight significant change in the extent to which adults seeking help to stop smoking in the UK are asking about e-cigarettes: 91% of responding practitioners in 2013 had experienced an increase in queries from smokers. The proportion of respondents who had no clients regularly using e-cigs fell from 19% to 4%. This reflects the increasing number of people using e-cigarettes in the UK in this period, which has also been reported elsewhere [2].

Questions were also included about e-cigarettes in the NCSCT SSS Practitioner Survey carried out between 4th December 2012 and 4th January 2013 (N = 1284) [16]. Identical questions were used to assess ever use and regular use. The modal category for ever use was ‘less than a quarter’ encompassing 52% of responses. In the 2011 survey reported here, this category included 80% of responses and in July 2013 this category included 43% of responses. Fifteen percent of respondents in the NCSCT Practitioner Survey reported that ‘a quarter to half of clients’ were regularly using e-cigarettes. In the 2011 and July 2013 surveys reported here the percentages were 2% and 23.5% respectively. Thus the NCSCT Practitioner Survey results were intermediate to the results of the two surveys reported here but slightly closer to the July 2013 survey, as expected. Results are expected to be close as the sampling frames were similar for both studies. However 21% of NCSCT Practitioner Survey respondents reported that they had no clients who regularly used e-cigarettes which is higher than both surveys reported here. This may reflect that the surveys reported here were only focused on e-cigarettes and so may have attracted more responses from practitioners whose clients were using e-cigarettes.

Currently most smoking cessation practitioners do not recommend e-cigarettes as cessation aids, because they are not licensed as medicines and therefore health service staff are not able to provide them [16]. Practitioners, however, do report clients using e-cigarettes for quitting and harm reduction [16]. In this current study the proportion holding positive views was higher in the second survey, perhaps reflecting the growing acceptance or visibility of e-cigarettes in UK society. Over a tenth of respondents who commented made supportive statements suggesting that they believed that e-cigarettes helped clients with cessation or harm reduction in terms of cutting down their smoking. In another study it was found that, despite only recruiting smokers who wanted to use e-cigarettes for harm reduction not cessation, 9% had quit by 52 weeks [29]; so there is some evidence that harm reduction with e-cigarettes may lead to abstinence from smoking. Respondents recommended that, in their view, further research and guidance are needed in order to enable practitioners to advise smokers about any risks, and about e-cigarettes’ potential role as an alternative to smoking. There was considerable uncertainty about existing current guidance in the UK and its implications.

Most responding practitioners were not positive about e-cigarettes in either survey; in particular they were perceived as promoting the continuation of smoking and challenging smoke free legislation. Marketing and advertising of combustible cigarettes has largely been banned and respondents raised concerns about the marketing of a product that resembled combustible cigarettes, although this was perhaps a reflection more of issues raised by the wider tobacco control community [30,31] rather than responding practitioners’ experiences with clients. There were, however, particular concerns raised in the comments about responding practitioners’ observations of the impact of e-cigarettes on young people. Some studies suggest that there is the potential for children to confuse e-cigarettes with combustible cigarettes [32], that e-cigarette use is growing fast [33] and that e-cigarette use is apparent among some young people who do not smoke combustible cigarettes [34]. Whether using e-cigarettes can become a gateway to using combustible cigarettes has not been established. More research on this is urgently required and the new UK legislation banning sales of e-cigarettes to children under the 18, announced in January 2014 [35] is likely to be welcomed.

There were also growing numbers of reports of SSS clients who had become dependent on e-cigarettes. Elsewhere [36] it has been found that 89% of e-cigarette users continued to use e-cigarettes one year after they were first monitored. A recently published study has found that it is possible for e-cigarette users to get as much nicotine from e-cigarettes as from combustible cigarettes [37] which heightens the likelihood of dependence occurring. However this also heightens the chances of e-cigarettes being effective for cessation. Furthermore nicotine in regulated nicotine products is not a harmful substance [38] although varying levels of nicotine in unregulated e-cigarettes may have consequences [8] and the effects of inhaling e-cigarette vapour in indoor air, particularly over a sustained period, are not yet fully understood [39,40].

Responding practitioners had concerns that potential quitters were bypassing licensed cessation quitting aids in favour of e-cigarettes. Nevertheless the survey suggest that smokers using e-cigarettes still approached the SSS suggesting such clients believed they required more than just e-cigarettes to achieve success in their quit attempt. In 2012/13 the number of clients accessing the SSS fell slightly for the first time in a number of years, but the proportion who successfully quit rose [23]. It is not possible to ascertain whether e-cigarettes were responsible for either of these changes. International data is also unclear on whether e-cigarettes do provide a clear cessation advantage, although research in this area is growing rapidly [6,41,42].

### Limitations

Our conclusions are limited by the small sample size and the need to rely on practitioners’ recall of clients’ stated experiences which adds layers of removal from actual events. It was not possible to know how much practitioners’ opinions were shaped by what they had heard through the media or tobacco control networks or whether what they reported was limited to their direct experience of working with clients. Furthermore, clients’ descriptions of their usage of e-cigarettes may have variations in accuracy. There are also statistical limitations: the chi square tests were conducted as if the two samples were independent - however it is likely that some respondents completed both surveys.

New questions were developed for these surveys and some issues arose with a couple of these. In 2011 clients were only able to indicate one query type they had received from clients whereas in 2013 they could indicate as many as applied. This meant that we could not statistically compare the queries from two surveys. The question “Are e-cigarettes a ‘good thing?’” has ambiguities as practitioners could be comparing e-cigarettes to combustible cigarettes or to not smoking at all. The complexities of this issue become apparent when inspecting the verbatim comments.

It is not possible to calculate the response rate because the total number of practitioners in the UK is unknown [16]. However commissioners in England now tend to make registration with NCSCT mandatory so it is likely that the NCSCT by 2013 had registered all English practitioners.

Given the higher reported client use of e-cigarettes by practitioners in the sample compared with practitioners generally [16], it is likely that the results are affected by some response bias. Thus readers should be wary of generalising the results reported here to all practitioners.

In addition, this dataset was not sufficient to be able to understand the implications of the MHRA decision in the UK - to do so it would be necessary to have time series data and to take into account other changes in marketing and smoking prevalence. Findings from the UK may not necessarily be applicable to other countries particularly where e-cigarette sales are more restricted.

## Conclusions

The surveys suggest that there is substantial interest in using e-cigarettes to support quit attempts among smokers seeking help from smoking cessation services and that this interest is growing. The results also indicate that these smokers are asking cessation practitioners a range of important questions about these products. The quantitative data and comments at both time points however, showed lack of consensus among practitioners on whether e-cigarettes are a positive or negative development and thus views of whether e-cigarettes have an important place in tobacco control and their impact on cessation services were inconsistent. Despite the MHRA announcement, practitioners in 2013 continued to ask for more research and information so that effective guidance for smoking treatment service clients and their staff can be put in place. Randomized controlled trials and ongoing surveillance of e-cigarette use and its consequences for smoking cessation rates and smoking treatment services are required.

## Abbreviations

EU: European union; MHRA: Medicines healthcare regulatory agency; NCSCT: UK national centre for smoking cessation and training; NHS: UK national health service; NRT: Nicotine replacement therapy; SSS: UK NHS Stop Smoking Services; UK: United Kingdom of Great Britain and Northern Ireland; US: United States of America.

## Competing interests

Maciej L. Goniewicz has received research funding from *Pfizer*, a manufacturer of stop smoking medication.

Andy McEwen receives a personal income from Cancer Research UK via University College London. He has received travel funding, honorariums and consultancy payments from manufacturers of smoking cessation products (Pfizer Ltd, Novartis UK and GSK Consumer Health care Ltd). He also receives payment for providing training to smoking cessation specialists, receives royalties from books on smoking cessation and has a share in a patent of a nicotine delivery device.

## Authors’ contributions

RH was involved in the quantitative analysis, undertook the majority of the verbatim data analysis and contributed substantively to the drafting of the article. MLG, AMc, LB, DA, and MD conceived of the study and participated in the design of the study and survey items. MLG wrote the first draft of the introduction and added substantially to the literature review and undertook the initial coding of the 2011 survey verbatim comments. AMc heads the NCSCT and led the data collection. SM performed the statistical analysis, contributed to the verbatim coding and wrote the first draft of the methods and results. LB directed the quantitative analysis and wrote the first draft of the discussion and conclusions. All authors were involved in revising the manuscript for important intellectual content. All authors read and approved the final manuscript.

## Supplementary Material

Additional file 1 **Coding frame for verbatim data.** This Microsoft Excel file contains codes and subcodes and number of times each code arose in the first and second survey verbatim comments and what percentage of comments from each survey contained each code. Click here for file
